# Generation of Interleukin-2 Receptor Gamma Gene Knockout Pigs from Somatic Cells Genetically Modified by Zinc Finger Nuclease-Encoding mRNA

**DOI:** 10.1371/journal.pone.0076478

**Published:** 2013-10-09

**Authors:** Masahito Watanabe, Kazuaki Nakano, Hitomi Matsunari, Taisuke Matsuda, Miki Maehara, Takahiro Kanai, Mirina Kobayashi, Yukina Matsumura, Rieko Sakai, Momoko Kuramoto, Gota Hayashida, Yoshinori Asano, Shuko Takayanagi, Yoshikazu Arai, Kazuhiro Umeyama, Masaki Nagaya, Yutaka Hanazono, Hiroshi Nagashima

**Affiliations:** 1 Laboratory of Developmental Engineering, Department of Life Sciences, School of Agriculture, Meiji University, Kawasaki, Japan; 2 Meiji University International Institute for Bio-Resource Research (MUIIBR), Kawasaki, Japan; 3 Division of Regenerative Medicine, Center for Molecular Medicine, Jichi Medical University, Tochigi, Japan; 4 CREST, Japan Science and Technology Agency, Tokyo, Japan; Southern Illinois University School of Medicine, United States of America

## Abstract

Zinc finger nuclease (ZFN) is a powerful tool for genome editing. ZFN-encoding plasmid DNA expression systems have been recently employed for the generation of gene knockout (KO) pigs, although one major limitation of this technology is the use of potentially harmful genome-integrating plasmid DNAs. Here we describe a simple, non-integrating strategy for generating KO pigs using ZFN-encoding mRNA. The interleukin-2 receptor gamma (*IL2RG*) gene was knocked out in porcine fetal fibroblasts using ZFN-encoding mRNAs, and *IL2RG* KO pigs were subsequently generated using these KO cells through somatic cell nuclear transfer (SCNT). The resulting *IL2RG* KO pigs completely lacked a thymus and were deficient in T and NK cells, similar to human X-linked SCID patients. Our findings demonstrate that the combination of ZFN-encoding mRNAs and SCNT provides a simple robust method for producing KO pigs without genomic integration.

## Introduction

Pigs have attracted attention as large experimental animals capable of providing valuable information that is highly extrapolatable to humans due to their anatomical, physiological, and hematological features [1]–[5]. To date, pig models of various human diseases, such as cystic fibrosis [6], diabetes mellitus [7], [8], Alzheimer's disease [9], and retinitis pigmentosa [10], have been created. In addition, research on the use of genetically modified pigs as organ/tissue donors for xenotransplantation into humans is advancing [11], [12]. In fact, encapsulated porcine islets of Langerhans have been transplanted into humans and are now under clinical trials to assess their safety and efficacy for curing type I diabetes mellitus [13].

The knockout (KO) of endogenous genes is a useful tool for analyses of gene function and the production of animal models that mimic human diseases. A variety of gene KO mice have been generated using embryonic stem (ES) cells genetically modified by homologous recombination (HR). As authentic ES cells are not available in pigs, HR using somatic cells has been employed to generate gene KO pigs in combination with somatic cell nuclear transfer (SCNT) technology. However, the low efficiency (frequency, 10^−6^ to 10^−8^) of HR for mammalian cultured cells hinders the generation of KO pigs [14]–[16], and the generation of KO pigs through HR therefore remains limited.

One new technique uses zinc finger nucleases (ZFNs) to knock out endogenous genes and is expected to overcome the inefficiency and complexity of HR in mammals [17]. Engineered ZFNs are artificial restriction enzymes comprised of a zinc finger DNA-binding domain and a DNA cleavage domain [18]. We previously were the first to demonstrate that gene KO in primary porcine fetal fibroblasts *in vitro* was possible using ZFNs [19], and somatic cells that were genetically modified by ZFNs were shown to be capable of producing gene KO pigs after SCNT [20]–[23]. In these studies, the ZFN-encoding plasmid DNA was introduced into somatic cells or the nuclear donor cells for SCNT. However, plasmid DNA can also be integrated into the genome of cells, which may result in the disruption of endogenous genes and the constitutive expression of ZFNs. This drawback of plasmid DNA can be eliminated by the use of ZFN-encoding mRNA, which cannot be inserted into the host genome. Gene KO using ZFN-encoding mRNAs in rodents has been performed via direct injection into the fertilized eggs [24]–[26], although the generation of KO piglets using ZFN-encoding mRNA has yet to be reported.

The present study sought to investigate whether ZFN-encoding mRNAs can be used to generate gene KO pigs. We chose the interleukin-2 receptor gamma (*IL2RG*) gene on the X-chromosome of male cells as a target gene to be knocked out. *IL2RG* encodes the common gamma chain (γ_c_), and mutations in *IL2RG* lead to X-linked severe combined immunodeficiency (XSCID), which is characterized by profound defects in cellular and humoral immunity in humans [27], [28]. Furthermore, knockout of *IL2RG* was previously shown to give rise to the XSCID phenotype in male pigs [29]. We therefore applied ZFN-encoding mRNA to knock out *IL2RG* in male porcine fibroblast cells, which are capable of supporting the development to live offspring after SCNT. Here, we show that an endogenous gene in porcine primary cultured cells could be knocked out using ZFN-encoding mRNAs, thereby allowing the efficient production of a gene KO pig by means of somatic cell cloning.

## Results

### Design of ZFNs and isolation of *IL2RG* KO cells

**Figure 1:**
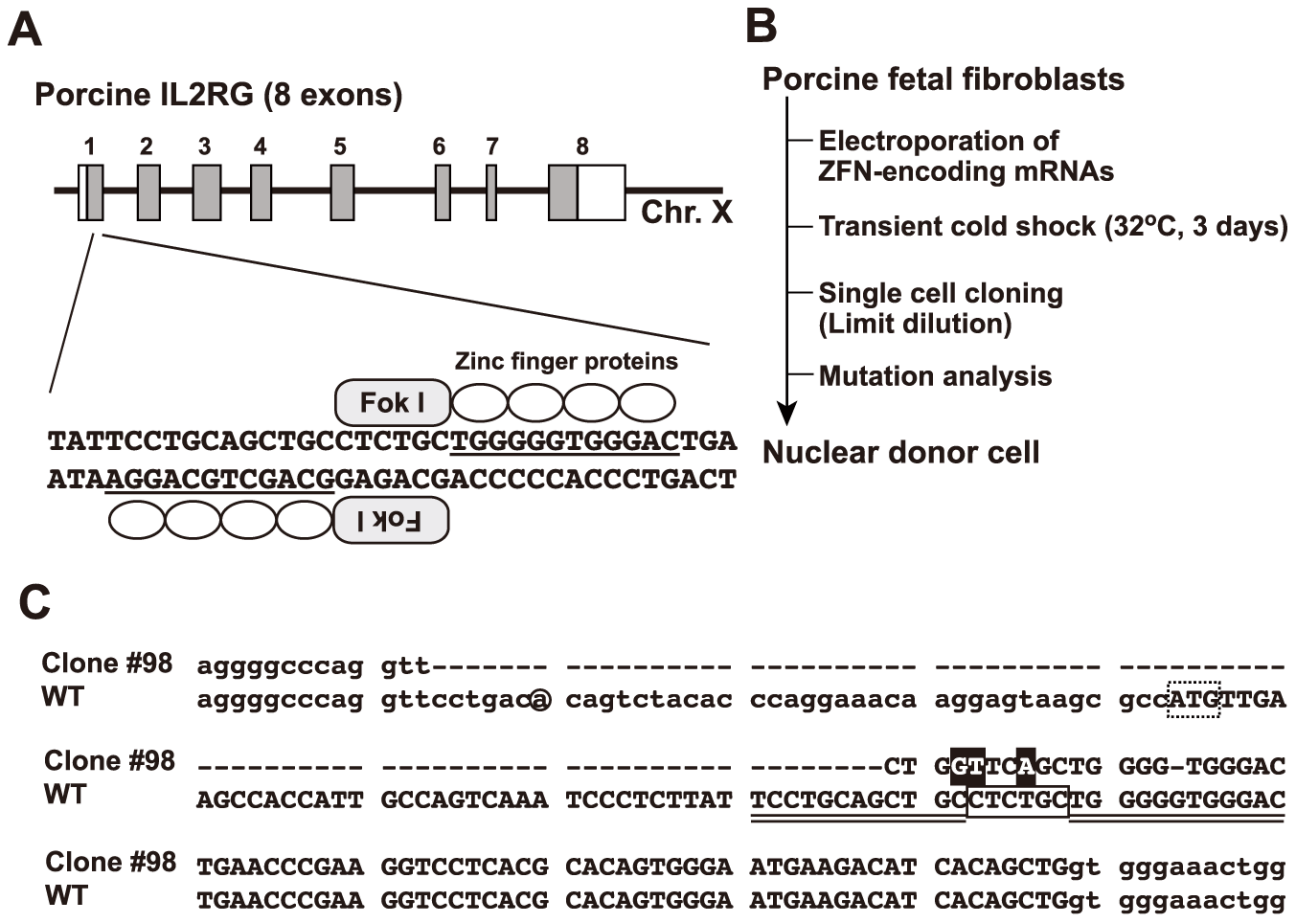
Design of ZFNs targeting the pig *IL2RG* gene and isolation of nuclear donor cells. (A) Schematic representation of ZFNs binding to pig *IL2RG*. The coding and untranslated regions are indicated by gray and white boxes, respectively. A ZFN consists of a nuclease domain (Fok I) and a DNA-binding domain (zinc finger proteins), and the recognition sequences of the zinc finger proteins are underlined. (B) Flow chart for the isolation of nuclear donor cells (clone #98) for SCNT. (C) ZFN-induced mutation in cell clone #98. The upper and lower sequences represent the WT and clone #98 sequence of *IL2RG*, respectively. The deletion mutation and nucleotide substitution in clone #98 are indicated by a hyphen and black box, respectively. The initiation codon of *IL2RG* is shown in a dotted box. The ZFN-binding and ZFN-cleavage sites are double-underlined and boxed, respectively. The major transcription initiation site is indicated with a circle.

Similar to *IL2RG* in humans, mice, and rats, porcine *IL2RG* is found on the X chromosome and consists of 8 exons [30]. In this study, we constructed a ZFN that targets exon 1 of porcine *IL2RG*. This pair (right and left) of ZFNs contains 4 zinc finger proteins each, and both the right and left ZFNs recognize a target sequence of 24 bp (Figure 1A). *IL2RG* KO cells were generated via the electroporation of ZFN-encoding mRNAs into porcine male fetal fibroblasts with transient cold shock treatment at 32°C for 3 d [31]. No visible morphological abnormalities were detected in the fetal fibroblasts following the introduction of mRNA and transient cold shock treatment. Of the 192 single cell-derived cell lines obtained by limiting dilution, 1 cell line (1/192, 0.5%) with a ZFN-induced mutation was established, and this cell line (#98, Figure 1B) was used as the nuclear donor for SCNT. DNA sequence analyses showed that these cells carried both a 3-bp substitution and an 86-bp deletion spanning the major transcription start point and the start codon (ATG) of porcine *IL2RG*, indicating that this mutation was likely to disrupt *IL2RG* function. Sufficient numbers of KO cells were prepared for SCNT after culture for 3 weeks.

### Production and analysis of *IL2RG* KO cloned pigs

**Figure 2:**
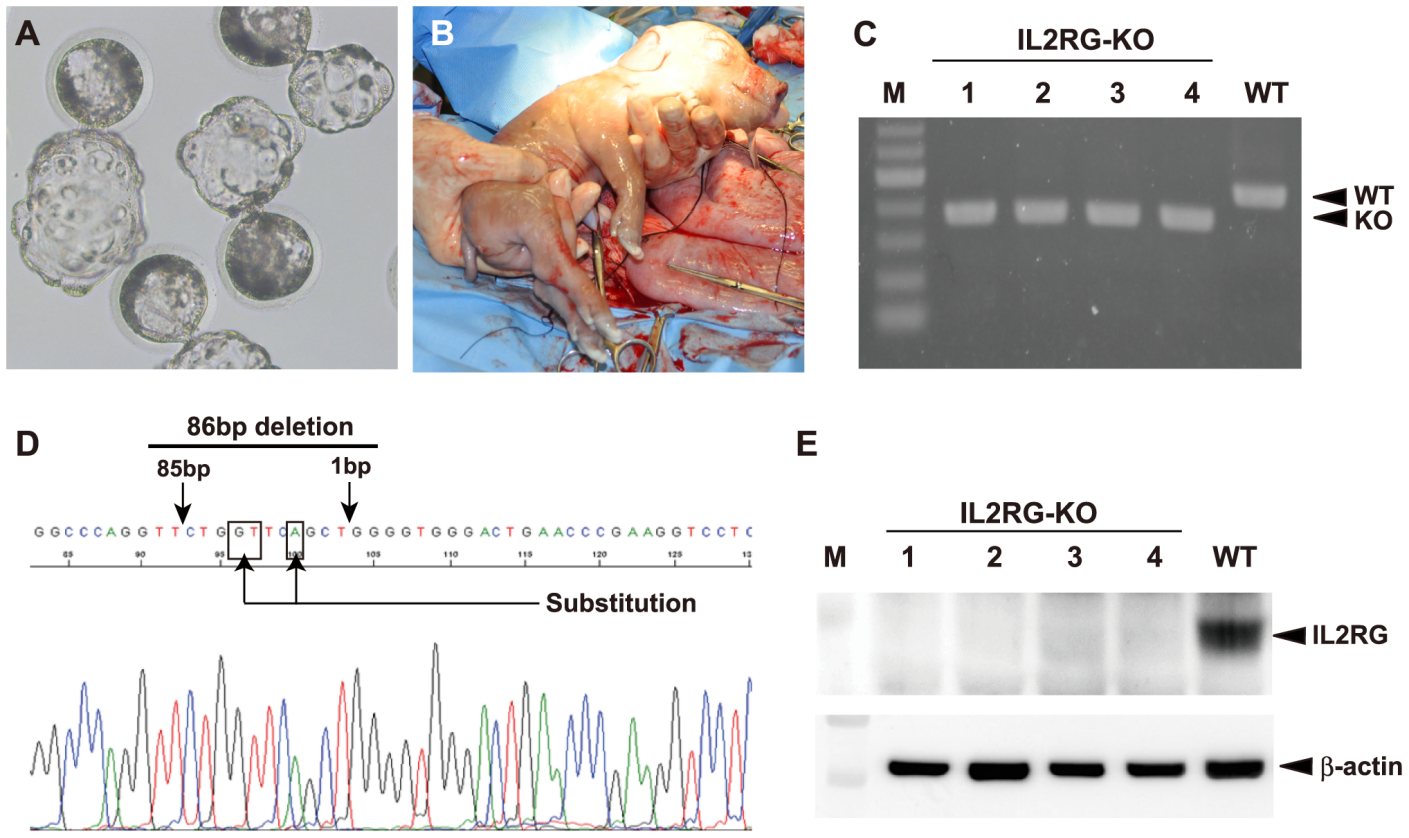
Generation and analysis of *IL2RG* KO pigs. (A) Cloned blastocysts transferred to recipient gilts. (B) Cloned *IL2RG* KO pig delivered by cesarean section at 113 d of gestation. (C) PCR genotyping for the 4 cloned piglets obtained. M: DNA marker. (D) The DNA sequence analysis of *IL2RG* in a cloned pig. The arrows and boxes indicate the same mutation as that of the nuclear donor cell (clone #98). (E) Western blot for IL2RG protein in the spleens of *IL2RG* KO pigs. β-actin was used as a loading control. M: protein standard marker.

First, the developmental competence of the SCNT embryos reconstructed with the *IL2RG* KO cells was examined *in vitro*. Of the 403 SCNT embryos produced in duplicated experiments, 237 (58.8%) developed into blastocysts (Table 1). This blastocyst formation rate was comparable to those reported in our previous studies [32]. Second, 199 blastocysts (Figure 2A) obtained by SCNT were subjected to transfer to 2 estrus synchronized recipient gilts (P177 and P178; Table 1). Pregnancy was confirmed in both gilts at 39 d of gestation. On day 113 of gestation, 4 male cloned pigs were obtained from 1 recipient (P177) via cesarean section (Figure 2B). The body weight and length of the 4 piglets ranged from 0.56 to 1.16 kg and 22 to 28 cm, respectively. The other recipient (P178) miscarried at 46 d of gestation.

**Table 1:** *in vitro* development of SCNT embryos and production of *IL2RG* KO pigs.

*in vitro* development of reconstructed SCNT embryos		
SCNT embryos reconstructed	403	
Normally cleaved embryos on day 2	151 (71.9%)	
Blastocyst-stage embryos on day 5	237 (58.8%)	
Production of *IL2RG* KO pigs		
Recipient	P177	P178
Blastocysts transferred^a^	100	99
Pregnancy	+	+
Cloned fetuses obtained	4 (4.0%)	- (miscarried)^b^

a Day 5–6 embryos.

b 46 d of gestation.

PCR genotyping and DNA sequence analyses of the 4 cloned pigs showed that all 4 pigs had the same mutation as the nuclear donor cells (3-bp substitution and 86-bp deletion; Figure 2C and D). Western blot analyses further showed that all 4 pigs lacked the IL2RG protein (Figure 2E).

### Phenotypic characterization of *IL2RG* KO pigs

**Figure 3:**
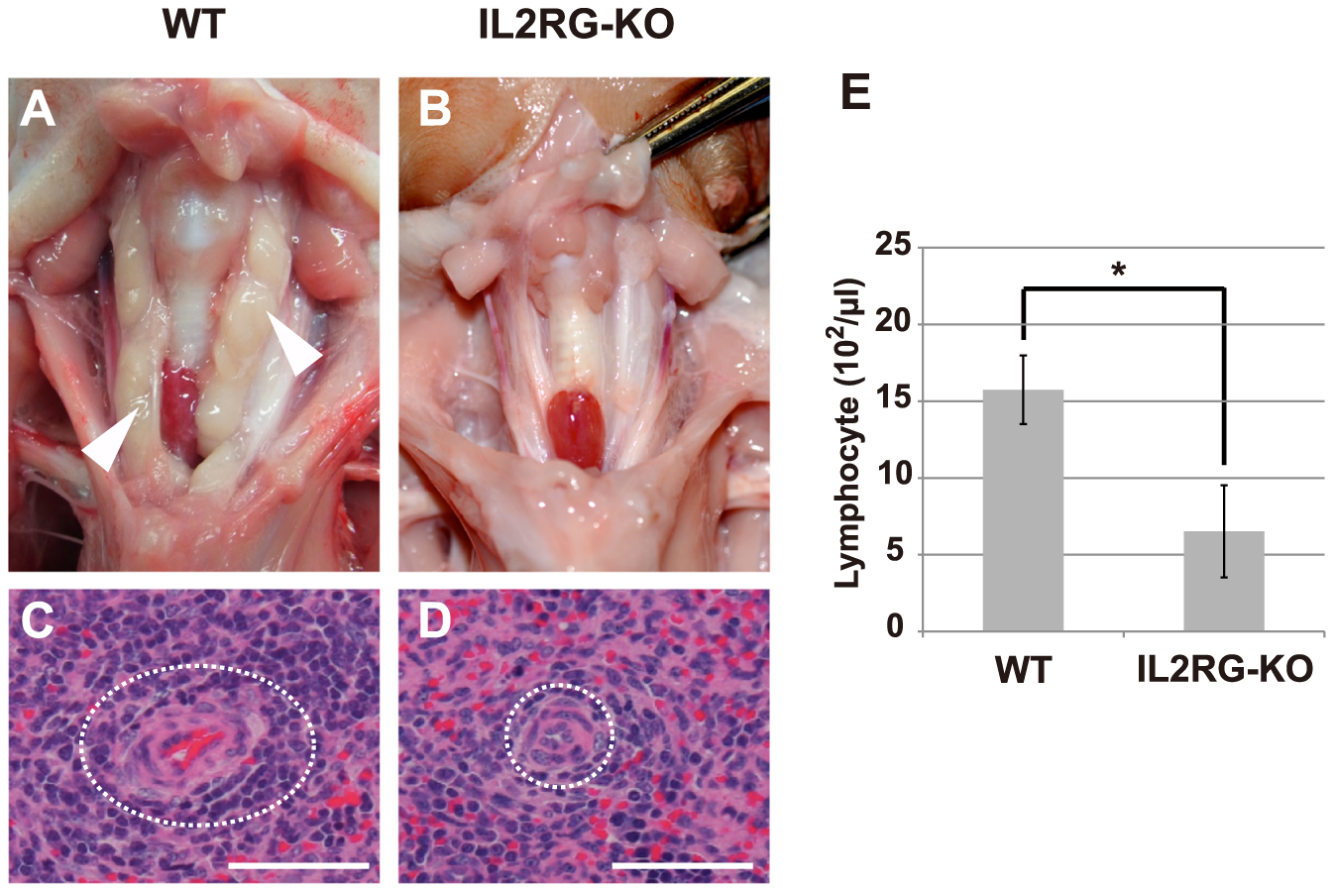
Phenotypes of *IL2RG* KO pigs. (A, B) The thymic phenotype in WT and *IL2RG* KO pigs. The white arrowheads indicate normal thymuses in WT pigs. (C, D) Histological analysis of the spleens of WT and *IL2RG* KO pigs. The white pulp of the spleen is indicated by a dotted white circle. Bar = 100 µm. (E) The proportion of lymphocytes in the peripheral blood (PB) of WT and *IL2RG* KO pigs. The data represent the means ± SD values for 4 pigs. The asterisk indicates a statistically significant difference (P<0.01) between the values for WT and *IL2RG* KO pigs (n = 4).

Gross anatomical analysis revealed that all 4 *IL2RG* KO pigs completely lacked thymuses (Figure 3A, B). Histological analysis of the spleens clearly showed the presence of lymphocytes in the white pulp of the peripheral lymphoid sheath tissue (PALS) in wild-type (WT) pigs (Figure 3C), whereas the *IL2RG* KO pigs showed very few or no lymphocytes in the PALS (Figure 3D). Embryonic hematopoiesis in the red pulp was strong in both WT and *IL2RG* KO pigs (data not shown). The lymphocyte counts in the peripheral blood of the WT and *IL2RG* KO pigs were 15.7±2.2×10^2^/ µl and 6.5±3.0×10^2^/ µl, respectively, indicating a significant reduction in the lymphocyte number in *IL2RG* KO pigs (P<0.01; Figure 3E).

**Figure 4:**
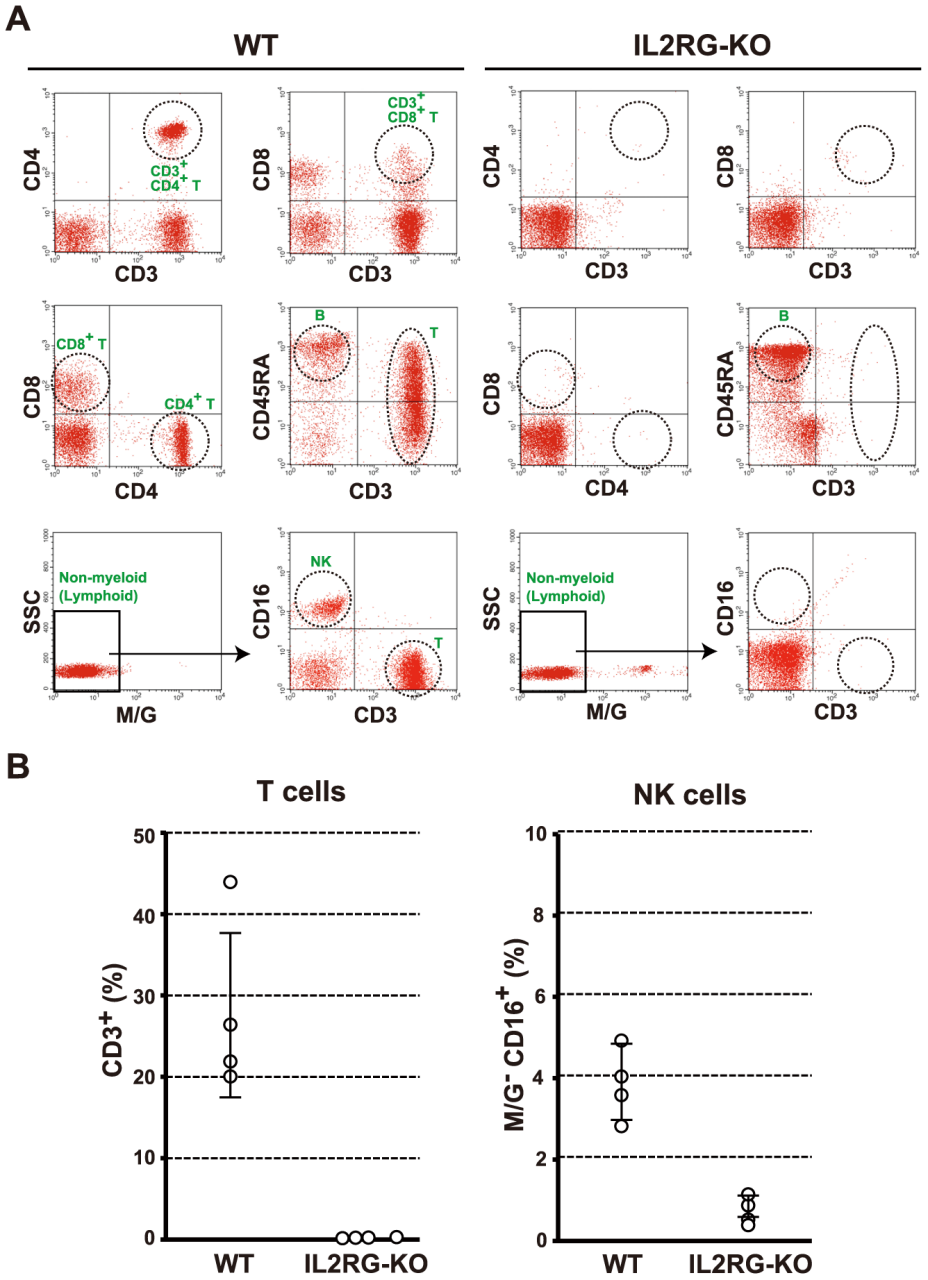
Flow cytometric analysis of mononuclear cells in *IL2RG* KO pigs. (A) Flow cytometric analysis of T, B, and NK cells in the peripheral blood of *IL2RG* KO pigs. The dot plots show CD3, CD4, and CD8 cells for the demarcation of T cell subpopulations and CD3, CD45RA, and CD16 (in the non-myeloid fraction, i.e., monocyte/granulocyte (M/G)-negative) cells for the differentiation of T cell, B cell, and NK cell subpopulations in the peripheral blood, respectively. (B) The proportion of T (CD3^+^) and NK (M/G^−^, CD3^−^, CD16^+^) cells among the mononuclear cells in the spleens of *IL2RG* KO pigs. The data represent the mean ± SD values of the 4 pigs obtained.

Flow cytometric analyses of the peripheral blood (Figure 4A) showed that the number of CD3^+^ T cells in *IL2RG* KO pigs (0.3%±0.1%) was drastically lower than that in WT pigs (74.0%±10.2%; P<0.0001). In addition, *IL2RG* KO pigs lacked CD3^+^CD4^+^ and CD3^+^CD8^+^ T cells. The number of NK cells (monocyte/granulocyte^−^, CD3^−^, and CD16^+^) was also notably lower in *IL2RG* KO pigs than WT pigs (*IL2RG* KO, 0.9%±0.2% vs. WT, 8.1±4.5%; P = 0.004), although the B cell population (CD3^−^ and CD45RA^+^) in *IL2RG* KO pigs was observed to be the same as that in WT pigs. As observed in the peripheral blood, the numbers of splenic T cells (*IL2RG* KO, 0.2%±0.1% vs. WT, 28.1%±10.9%; P<0.0001) and NK cells (*IL2RG* KO, 0.8%±0.3% vs. WT, 3.9%±0.8%; P = 0.0001) were significantly reduced in *IL2RG* KO pigs (Figure 4B). Thus, an almost complete lack of T and NK cells was observed in the *IL2RG* KO pigs, which is similar to human XSCID patients.

## Discussion

In rodents, the microinjection of ZFN-encoding mRNA into fertilized eggs has been used for the creation of gene KO animals, mainly due to its simplicity. However, the drawbacks of this microinjection method include inefficiency and the occurrence of mutation mosaicism [24]. The transfer of mRNA-injected eggs into recipient females gives rise to both non-mutant and mutant offspring, and the generation of mutants results in undesired mutations that are meaningless with regard to the traits of the gene KO animals. Mutation mosaicism can result from sustained ZFN activity during later embryogenesis or the re-cleavage of the already-modified alleles [33], [34]. Individuals with the desired mutation can be selected after crossbreeding with WT animals. Such a breeding process, however, requires enormous time, labor, and costs in large animals such as pigs, which have longer gestation intervals than rodents. We therefore applied the gene KO procedure using SCNT for the generation of *IL2RG* KO pigs in the present study. With this method, nuclear donor cells could be examined *in vitro* for the induced mutations prior to the production of cloned animals by SCNT [2]. Thus, the wasteful production of undesired animals can be avoided. To our knowledge, this study is the first to demonstrate the generation of cloned pigs from gene KO cells prepared using ZFN-encoding mRNA.

For the generation of gene KO pigs by somatic cell cloning, HR has traditionally been used to knock out a target gene in nuclear donor cells [11], [12], [29]. In HR, an antibiotic-based cell selection is performed to obtain KO cells; however, several issues arise, including (1) the insertion of an antibiotic cassette into the host genome using targeting vectors, (2) the senescence or exhaustion of nuclear donor cells caused by the prolonged culture associated with antibiotic selection, and (3) the unavoidable contamination of non-targeted cells despite the positive–negative screening [29], [35]–[37]. Therefore, a re-cloning process, namely repeated nuclear transfer, is often necessary to obtain KO offspring [38], [39]. In the re-cloning process, fetuses are collected after the first round of SCNT and embryo transfer, and these first-round cloned fetuses can be analyzed for gene KO status. The establishment of primary culture cells from the gene KO fetus requires obtaining rejuvenated nuclear donor cells for the next round of SCNT. Using these rejuvenated cells, the antibiotic cassette can be excised, provided that the proper site-specific recombinase technology, such as Cre-*lox*P recombination, was incorporated [40].

In contrast, ZFN-encoding mRNAs can generate gene KO cells without antibiotic selection. In fact, sufficient numbers of nuclear donor cells for SCNT can be obtained in a short period of time (approximately 3 weeks). Moreover, the *IL2RG*-KO cells generated by the ZFN-encoding mRNAs in this study allowed for the direct production of full-term cloned fetuses without rejuvenation of the nuclear donor cells and subsequent re-cloning. As a result, we obtained full-term cloned fetuses within 6 months, including the period spent establishing the KO cells, whereas the HR method requires an average of 12 to 18 months to obtain KO animals. An additional advantage of ZFN-encoding mRNAs is transient ZFN expression, which reduces the incidence of off-target mutations [41]. Off-target events are a potential limitation of the ZFN technique [26], [42], [43], although the introduction of ZFN-encoding mRNAs leads to the immediate translation of ZFNs in the cytoplasm without the risk of genomic integration, which could disrupt endogenous genes. Carlson et al. recently generated KO pigs using TALEN-encoding mRNA [44]. Based on these collective results, we believe that it is important to compare the efficiencies of ZFN- and TALEN-mRNA in generating KO pigs.

A marked decrease in the number of T and B cells has been reported in XSCID mice [45], [46] and rats [24]. In human XSCID patients, although the number of T and NK cells is significantly decreased, the number of B cells remains normal or is occasionally increased [28], [47]. Thus, the phenotypes of rodent XSCID models do not necessarily mimic the conditions of human XSCID. In contrast, the *IL2RG* KO pigs obtained in this study lacked T and NK cells but showed normal B cell populations, and identical phenotypic characteristics were shown in a previous report in which XSCID pigs were generated through HR [29]. Thus, *IL2RG* KO pigs are considered to be an accurate model that mimics human XSCID.

Opportunistic infections in XSCID animals after birth are unavoidable under conventional housing conditions. We therefore used the full-term *IL2RG* KO pig fetuses recovered via cesarean section (113 d of gestation) for our analyses to avoid any changes due to infections.

In conclusion, this study presents a simple, non-integrating strategy for generating KO pigs using ZFN-encoding mRNA, which successfully generated *IL2RG* KO pigs via the SCNT method in a short period of time. The combination of ZFN-encoding mRNA with SCNT provides a robust method for generating KO pigs without genomic integration. Moreover, the resulting *IL2RG* KO pigs showed a phenotype similar to that of human XSCID. Although further characterization is required, these findings represent the first step toward developing a porcine SCID model, and we believe that this *IL2RG* KO pig model will greatly contribute not only to cancer and stem cell research but also to preclinical evaluations of the transplantation of pluripotent stem cells, such as iPS cells.

## Materials and Methods

### Animal care and chemicals

All of the animal experiments in this study were approved by the Institutional Animal Care and Use Committee of Meiji University (IACUC10-0004). All chemicals were purchased from the Sigma-Aldrich Chemical Co. (MO, USA) unless otherwise indicated.

### Design of ZFNs and mRNA preparation

Custom ZFN plasmids for pig *IL2RG* were obtained from Toolgen Inc. The design and validation of these ZFNs was performed by Toolgen Inc (Seoul, South Korea). The constructed ZFNs were designed to target the sequence of exon 1 in the pig *IL2RG* gene. Each of the ZFNs had 4 zinc finger domains recognizing 12 bases (Figure 1). For the production of ZFN-encoding mRNA, each of the ZFN plasmids was digested with the restriction enzyme Xho I. The linearized plasmids were then purified with phenol/chloroform to generate a high-quality DNA template for *in vitro* transcription. Capped ZFN mRNA was produced from the linearized DNA template via *in vitro* transcription using a MessageMAX T7 ARCA-Capped Message Transcription Kit (Cambio, Cambridge, UK). A poly(A) tail was then added to each mRNA by polyadenylation using the Poly(A) Polymerase Tailing Kit (Cambio). The poly(A)-tailed ZFN-encoding mRNA was then purified using a spin column with the MEGAclear Kit (Life Technologies, CA, USA) and finally resuspended in RNase-free water at 400 ng/ µl.

### Isolation of *IL2RG* KO cells and culture conditions

A primary culture of porcine fetal fibroblast cells (male line) was used as the progenitor line for the isolation of *IL2RG* KO cells. The fibroblast cells and their derivatives (KO cells) were seeded onto type I collagen-coated dishes or plates (Asahi Glass, Tokyo, Japan) and cultured in MEMα (Life Technologies) supplemented with 15% FBS (Nichirei Bioscience, Tokyo, Japan) and 1× antibiotic-antimycotic solution (Life Technologies) in a humidified atmosphere containing 5% CO_2_ at 37°C. The fetal fibroblasts were cultured to 70–90% confluence, washed twice with D-PBS(−) (Life Technologies), and treated with 0.05% trypsin-EDTA (Life Technologies) to isolate and collect the cells. The cells (4×10^5^) were then suspended in 40 µl of R buffer (supplied as part of the Neon Transfection System, Life Technologies), and 2 µl of ZFN-encoding mRNA solution (400 ng/ µl) was added. The cells were then electroporated under the following conditions: pulse voltage, 1,100 V; pulse width, 30 ms; and pulse number, 1 (program #6). Following electroporation, the cells were cultured at 32°C for 3 d (transient cold shock) first without antibiotics in the medium described above for 24 h and then with antibiotics in the medium [31]. For recovery after the transient cold shock treatment, the cells were cultured at 37°C until they approached confluence, and then limiting dilution was performed to obtain single cell-derived clones in five 96-well plates. At 12 d after limiting dilution, cells at relatively high confluency (>50%) in each well were selected and divided for further culture and mutation analysis. The cells at low confluency (∼50%) after limiting dilution were not used in further experiments.

### Analysis of ZFN-induced mutations in nuclear donor cells and cloned fetuses

The target region of IL2RG-ZFNs was amplified by direct PCR from the cell clones using MightyAmp DNA polymerase (Takara Bio, Shiga, Japan) and the corresponding primers (5′-ATAGTGGTGTCAGTGTGATTGAGC and 5′-TACGAACTGACTTATGACTTACC). Nested PCR was then performed using PrimeSTAR HS DNA polymerase (Takara Bio) and the appropriate primers (5′-ATACCCAGCTTTCGTCTCTGC and 5′-TTCCAGAATTCTATACGACC). Subsequently, the PCR fragment including the ZFN target region was examined using the sequencing primer 5′-AGCCTGTGTCATAGCATAC, the BigDye Terminator Cycle Sequencing Kit, and an ABI PRISM 3100 Genetic Analyzer (Life Technologies). For analysis of the mutation in cloned fetuses, genomic DNA was extracted from the tail biopsies of fetuses using a DNeasy Tissue and Blood Kit (QIAGEN, Hilden, Germany), and then PCR genotyping and DNA sequencing were performed as described above. All new sequence data is deposited in DDBJ/EMBL/GenBank (AB846644-AB846648).

### SCNT and embryo transfer

SCNT was performed as described previously with slight modifications [32]. Briefly, *in vitro*-matured oocytes containing the first polar body were enucleated via the gentle aspiration of the polar body and the adjacent cytoplasm using a beveled pipette in 10 mM HEPES-buffered Tyrode lactose medium containing 0.3% (w/v) polyvinylpyrrolidone (PVP), 0.1 µg/ml demecolcine, 5 µg/ml cytochalasin B (CB), and 10% FBS. Fibroblasts (clone #98) were used as nuclear donors following cell cycle synchronization via serum starvation for 2 d. A single donor cell was inserted into the perivitelline space of an enucleated oocyte. The donor cell-oocyte complexes were placed in a solution of 280 mM mannitol (Nacalai Tesque, Kyoto, Japan) (pH 7.2) containing 0.15 mM MgSO_4_, 0.01% (w/v) PVA, and 0.5 mM HEPES and were held between 2 electrode needles. Membrane fusion was induced with a somatic hybridizer (LF201; NEPA GENE, Chiba, Japan) by applying a single direct-current (DC) pulse (200 V/mm, 20 µs) and a pre- and post-pulse alternating current (AC) field of 5 V at 1 MHz for 5 s. The reconstructed embryos were cultured in NCSU23 medium supplemented with 4 mg/ml BSA for 1 to 1.5 h, followed by electrical activation. The reconstructed embryos were then washed twice in an activation solution containing 280 mM mannitol, 0.05 mM CaCl_2_, 0.1 mM MgSO_4_, and 0.01% (w/v) PVA and were aligned between 2 wire electrodes (1.0 mm apart) of a fusion chamber slide filled with the activation solution. A single DC pulse of 150 V/mm was applied for 100 µs using an electrical pulsing machine (Multiporator; Eppendorf, Hamburg, Germany). After activation, the reconstructed embryos were transferred into PZM5 supplemented with 5 µg/ml CB and 500 nM Scriptaid for 3 h. The embryos were then transferred into PZM5 supplemented with Scriptaid and further cultured for 12 to 14 h. After incubation, the embryos were further cultured in PZM5, and the dish was maintained under a humidified atmosphere of 5% CO_2_, 5% O_2_, and 90% N_2_ at 38.5°C. Beyond the morula stage, the embryos were cultured in PZM5 supplemented with 10% FBS.

Crossbred (Large White/Landrace × Duroc) prepubertal gilts weighing 100 to 105 kg were used as recipients of the SCNT embryos. The gilts were given a single intramuscular injection of 1,000 IU of eCG to induce estrus. Ovulation was induced by an intramuscular injection of 1,500 IU of hCG (Kawasaki Pharmaceutical, Kanagawa, Japan) that was given 66 h after the injection of eCG. The SCNT embryos cultured for 5 to 6 d were surgically transferred into the oviducts of the recipients approximately 146 h after hCG injection.

### Western blot analysis

After the *IL2RG* KO and age-matched WT pigs were sacrificed, their dissected spleens were homogenized in RIPA buffer (Thermo Scientific, MA, USA) with a protease inhibitor cocktail (Nacalai Tesque) and subjected to centrifugation, and the supernatants were collected. The protein concentrations of the samples were quantified using a DC protein assay (Bio-Rad, CA, USA) based on the Lowry method. Approximately 40 µg of protein from the spleen extracts was subjected to 10% SDS-PAGE and transferred by electroblotting to a Hybond-P PVDF membrane (GE Healthcare Bio-Sciences, NJ, USA). The membranes were blocked for 30 min at room temperature with Blocking One (Nacalai Tesque). After blocking, the membranes were incubated with an anti-IL2RG antibody (1∶200 dilution; Santa Cruz Biotechnology, CA, USA) for 1 h at room temperature and were subsequently incubated with HRP-conjugated anti-rabbit IgG antibody (1∶5,000 dilution; Santa Cruz Biotechnology) for 1 h at room temperature. The blot was developed using ECL Western Blotting Detection Reagents (GE Healthcare Bio-Sciences). The signal was detected and imaged with an ImageQuant LAS-4000 system (GE Healthcare Bio-Sciences).

### Flow cytometric analysis

Peripheral blood mononuclear cells were harvested from the whole blood and spleens of *IL2RG* KO pigs using the erythrocyte lysis solution PharmLyse (Becton Dickinson, BD, NJ, USA), and 1×10^6^ cells were incubated with mouse anti-pig CD3e (Abcam, Cambridge, UK), CD4a (BD), CD8a (BD), CD16 (AbDSerotec, NC, USA), CD45RA (AbDSerotec), and monocyte and granulocyte (M/G, Abcam) antibodies for 30 min at room temperature. After incubation, the cell suspension was washed and resuspended with PBS (−) supplemented with 1% FBS (w/v). The cell populations isolated from the peripheral blood and spleens of *IL2RG*-KO pigs were evaluated using a FACSCalibur flow cytometer (BD) equipped with a 488-nm argon laser. The cell debris and aggregates were gated out of the analysis using bivariate, forward/side scatter (FSC/SSC) parameters. In all analyses, the virtual lymphocyte population was gated, and the gated 1×10^4^ events per sample were acquired and analyzed using CELLQuest Pro software (BD).

### Histological analysis

After the *IL2RG* KO and age-matched WT pigs were sacrificed, their dissected spleens were fixed in 10% neutral buffered formalin solution (Wako Pure Chemical Industries, Osaka, Japan), embedded in paraffin, sectioned, and stained with hematoxylin and eosin using standard methods.
