# Correction: Generation of Interleukin-2 Receptor Gamma Gene Knockout Pigs from Somatic Cells Genetically Modified by Zinc Finger Nuclease-Encoding mRNA

**DOI:** 10.1371/annotation/99b0e7bf-5878-42de-bcce-719ceea15466

**Published:** 2014-01-07

**Authors:** Masahito Watanabe, Kazuaki Nakano, Hitomi Matsunari, Taisuke Matsuda, Miki Maehara, Takahiro Kanai, Mirina Kobayashi, Yukina Matsumura, Rieko Sakai, Momoko Kuramoto, Gota Hayashida, Yoshinori Asano, Shuko Takayanagi, Yoshikazu Arai, Kazuhiro Umeyama, Masaki Nagaya, Yutaka Hanazono, Hiroshi Nagashima

There are multiple errors in the "SCNT and embryo transfer" section of the Materials and Methods, and a value and percentage given in Table 1 is incorrect. Please see a corrected version of the aforementioned section and the correct value and percentage here: http://plosone.org/corrections/pone.0076478.cn.docx

